# A Multilevel Analysis of HIV Care Outcomes Across Age, Race, and Housing Among United States Women Veterans

**DOI:** 10.3390/healthcare14121781

**Published:** 2026-06-20

**Authors:** Giselle Day, Amber B. Amspoker, Alan Z. Sheinfil, Liang Chen, Emmanuel Guajardo, Maria E. Fernandez, Cici Bauer, Irene Tamí-Maury, Jan Lindsay

**Affiliations:** 1 Houston VA HSR&D Center for Innovations in Quality, Effectiveness and Safety, Michael E. DeBakey VA Medical Center, Houston, TX 77030, USA; aamspoker@central.uh.edu (A.B.A.); a.sheinfil@fdu.edu (A.Z.S.); liang.chen@va.gov (L.C.); jan.lindsay2@va.gov (J.L.); 2 VA South Central Mental Illness Research, Education and Clinical Center, Michael E. DeBakey VA Medical Center, Houston, TX 77030, USA; 3 College of Medicine, Houston, University of Houston, Houston, TX 77204, USA; 4 Humana Integrated Health Systems Sciences Institute, Houston, TX, 77204, USA; 5 Marion Turpan College of Psychology & Counseling, Fairleigh Dickinson University, Teaneck, NJ 07666, USA; 6 Menninger Department of Psychiatry and Behavioral Sciences, Baylor College of Medicine, Houston, TX 77030, USA; 7 Department of Medicine, Section of Health Services Research, Baylor College of Medicine, Houston, TX 77030, USA; 8 Southeast Louisiana Veterans Health Care System, New Orleans, LA 70119, USA; emmanuel.guajardo@va.gov; 9 Department of Medicine, Tulane University School of Medicine, New Orleans, LA 70112, USA; 10 Department of Health Promotion and Behavior Sciences, University of Texas Health Science Center at Houston (UTHealth Houston) School of Public Health, Houston, TX 77030, USA; maria.e.fernandez@uth.tmc.edu; 11 Department of Biostatistics and Data Science, University of Texas Health Science Center at Houston (UTHealth Houston) School of Public Health, Houston, TX 77030, USA; cici.x.bauer@uth.tmc.edu; 12 Department of Epidemiology, University of Texas Health Science Center at Houston (UTHealth Houston) School of Public Health, Houston, TX 77030, USA; irene.m.tami-maury@uth.tmc.edu

**Keywords:** HIV, women veterans, intersectionality, housing instability

## Abstract

**Highlights:**

**What are the main findings?**

- Among women veterans with HIV in VHA care, younger age and being unhoused were associated with lower odds of receiving care, being retained in care, and achieving viral suppression.
- Hispanic and non-Hispanic Black women veterans had better predicted HIV care outcomes than civilian population trends would suggest.

**What are the implications of the main findings?**

- VHA’s integrated health system may buffer some access barriers; system-level interventions are still needed to support younger and unhoused women veterans.
- MAIHDA is a feasible and informative analytic framework for examining intersectional disparities in VHA research.

**Abstract:**

Background: Women veterans with HIV represent a growing, diverse population within the Veterans Health Administration (VHA). This study examined HIV care continuum outcomes among women veterans using a multilevel intersectional approach. Methods: We conducted a retrospective analysis of 1154 women veterans with HIV in VHA care in fiscal year 2022, with outcomes assessed in fiscal year 2023. We applied Multilevel Analysis of Individual Heterogeneity and Discriminatory Accuracy (MAIHDA) to evaluate how combinations of social positions (age, race/ethnicity, housing status) were associated with HIV outcomes. Results: MAIHDA models showed that younger age (<45 years) and being unhoused were consistently associated with lower odds of care engagement and viral suppression than midlife (45–64) and older (65+) housed women veterans. Additionally, predicted probability analyses revealed distinct clustering patterns. Younger non-Hispanic White women consistently ranked among the lowest performing strata across all outcomes, while midlife and older Hispanic and non-Hispanic Black women veterans clustered among the highest. Variance Partition Coefficients from null models were modest (1.8–3.0%). Fully adjusted models showed no remaining between-stratum variance, suggesting that the included social positions explained the observed differences in our dataset. Conclusions: These findings highlight disparities in HIV care engagement concentrated among specific groups and reinforce the importance of addressing individual- and system-level barriers to engagement and continuity of care among women Veterans in VHA care.

## 1. Introduction

Women veterans are a rapidly growing, increasingly diverse population within the Veterans Health Administration (VHA) [1]. The VHA is the U.S.’ federal health system serving military veterans and is the largest integrated healthcare system in the country. Among women veterans, those with HIV face a range of unique healthcare challenges influenced by demographic and social factors, including age, race/ethnicity, and housing status [2,3]. Although national HIV care outcomes have greatly improved in recent years, with 70.3% viral suppression among all veterans in VHA care in 2022, women still face a higher risk of care gaps and lower viral suppression rates (62.3%) [4]. This may reflect a combination of biomedical factors (e.g., antiretroviral side-effects and menopause) and structural barriers (e.g., internalized and interpersonal stigma) [5,6].

Prior analyses of women veterans with HIV have shown patterns that diverge from civilian populations. While national civilian data show lower viral suppression rates among Black women (55.5%) than White women (59.6%), a VHA-based cohort found the opposite pattern with slightly higher viral suppression among Black (59.5%) than White (54.6%) women veterans [2]. This suggests that the VHA’s integrated care model may mitigate some barriers experienced by Black women veterans. However, many questions remain about which factors are most strongly associated with HIV care engagement and viral suppression among women veterans and how these factors interact.

Age is an especially important dimension of HIV care. Within VHA, retention in care and viral suppression improve with increasing age, likely reflecting life-stage factors, such as competing work and caregiving demands, health literacy differences, or variations in social support systems, as well as greater experience incorporating HIV care behaviors into daily life [4]. Housing instability often disrupts care engagement and continuity, and is associated with poorer outcomes, including lower antiretroviral therapy (ART) adherence and reduced viral suppression [7].

This study applies an intersectionality framework, which describes how interlocking systems and structures of power shape individuals’ experiences across multiple social positions to better capture these complexities [8,9,10]. Traditional analytic approaches that model these factors independently may overlook patterns affecting those who occupy multiple marginalized positions [11]. By moving beyond a single-axis framework (e.g., sex or veteran status or race only), this study aims to provide a more comprehensive understanding of disparities in HIV care engagement among women veterans.

Therefore, we applied the Multilevel Analysis of Individual Heterogeneity and Discriminatory Accuracy (MAIHDA) approach, which enables the modeling of combined social positions [12]. By providing a clearer picture of how intersecting social positions are associated with HIV outcomes in the VHA, we can inform tailored clinical interventions and broader system-level strategies.

## 2. Materials and Methods

### 2.1. Theoretical Framework

This study, guided by an intersectionality framework, employed a combination of McCall’s inter- and intracategorical approaches to intersectional complexity [8,9,10,13]. This approach uses existing social categories (intercategorical) to describe and analyze within-group variation (intracategorical). In this study, age captures life-course variation and generational context; race and ethnicity are conceptualized within social stratification and historical racial inequities in healthcare; and housing status is included as a marker of structural vulnerability (e.g., poverty and barriers to consistent care) and housing insecurity [14,15,16]. These variables are interpreted as a proxy for social positions that reflect broader structural conditions influencing access to and engagement with HIV care, rather than as independent risk factors.

### 2.2. Study Design and Data Source

We conducted a retrospective cohort analysis using national data from the Corporate Data Warehouse, a national electronic health records database maintained by the Veterans Affairs (VA) Information Resource Center and monitored for accuracy and validity. Analyses were performed using R Statistical Software (v4.4.2) [17]. This study was approved by Baylor College of Medicine’s institutional review board (H-54768) and the Michael E. DeBakey Veterans Affairs Medical Center Research and Development Committee.

### 2.3. Study Population

We extracted a national cohort of VA-enrolled women veterans with HIV in US federal fiscal year 2022 (FY22; October 2021–September 2022). We included women if they had at least one encounter in a VA Medical Center or an affiliated VA community-based outpatient center in FY22. HIV status was identified if women met at least two of the following criteria: (1) International Classification of Diseases, Tenth Revision (ICD-10) code for HIV (B20), (2) documented positive HIV laboratory test, or (3) an HIV ART prescription in the electronic health records [18].

### 2.4. Measures

Demographic variables were *age* (categorized: <45, 45–64, and 65+) to reflect life-stage differences; race/ethnicity (non-Hispanic Black, non-Hispanic White, and Hispanic, any race); and housing status (housed, unhoused) as predictors. To ensure stable estimates across strata, the analytic sample was limited to women whose race and ethnicity fell into these three categories. Smaller racial groups (n < 15) were excluded, and Hispanic individuals were modeled as a distinct category, recognizing that this approach masks heterogeneity among underrepresented populations. Housing status was defined based on documentation of care received in VHA clinics designated for homeless services, which serve as an administrative marker of homelessness or housing instability. These clinics are designated using VHA-specific billing codes that identify a veteran encounter with a clinic that provides targeted support to veterans experiencing housing instability or homelessness [19]. Our dataset does not include variables that distinguish homelessness from unstable housing. Therefore, we use “unhoused” as an umbrella term throughout, with the understanding that this obscures meaningful variation within this group.

Outcomes were three standard HIV care continuum indicators assessed in fiscal year 2023 (FY23; October 2022–September 2023): receipt of care (at least 1 HIV-related lab in FY23), retention in care (≥2 HIV-related labs at least 90 days apart in FY23), and viral suppression among all women in care (last test showing <200 HIV RNA copies/mL of blood in FY23). Outcomes were dichotomized (yes, no) based on whether they met definition criteria and are concordant with VHA administrative data-collection methods.

### 2.5. MAIHDA Rationale

We employed MAIHDA to model outcomes across multiple social positions with greater precision than traditional methods and because it is theoretically aligned with intersectionality frameworks [12,20,21]. MAIHDA allows individuals (level 1) to be nested within intersectional strata (level 2, i.e., older Hispanic women who are unhoused), with the assumption that women within the same stratum may share similar experiences navigating their HIV care within the VHA. In our analysis, up to 18 strata (3 × 3 × 2) can be generated based on all possible combinations of three age groups, three race/ethnicity categories, and two housing status groups.

We used temporally ordered data, with baseline characteristics (age, race/ethnicity, and housing status) measured in FY22 and HIV care outcomes (receipt, retention, and suppression) assessed in FY23. Although the data reflect temporal ordering, we employed a cross-sectional MAIHDA framework to model associations across intersecting social positions rather than a longitudinal approach with repeated measures. This analytic strategy is consistent with prior applications of MAIHDA in temporally ordered cohort data [22]. It also aligns with established multilevel modeling approaches to examine individual and contextual predictors of health outcomes [12,23]. MAIHDA offered several methodological advantages for our study, including robust precision-weighted estimates, avoiding collinearity and overfitting, simpler interpretability, and a comparison of additive versus multiplicative effects [24,25,26,27,28].

This analytic approach is especially valuable for identifying which groups face unique barriers or advantages, helping inform tailored interventions and system-level improvements. Importantly, MAIHDA was selected not because of the number of strata, but because it provides a principled framework to quantify whether disparities are attributed to additive main effects versus residual intersectional clustering.

### 2.6. Statistical Analysis

We fit two logistic regression MAIHDA models for each HIV outcome: a null MAIHDA model excluding main effects, and a full MAIHDA model including all main-effect covariates. We reported odds ratios (ORs) with their 95% confidence intervals (CIs).

#### 2.6.1. Null Model

For each outcome, we fit a null model, excluding individual-level covariates. These models provided baseline (unadjusted) stratum-level variation estimates and generated unique predicted probabilities for each stratum. We calculated the Variance Partition Coefficient (VPC) which provides an estimate of how much of the variation in the outcome is attributable to differences between strata. A high VPC suggests that individuals within the same stratum have greater similarity. Conversely, a low VPC indicates that most outcome variation occurs within the strata, suggesting that more individualized rather than group-level approaches may be needed [24].

#### 2.6.2. Full Models

We then fit full models, including all main-effect covariates and the random stratum intercept. We compared the VPC between the null and the full models to assess whether observed inequities followed primarily additive or multiplicative interaction patterns. Lastly, we calculated the Proportional Change in Variance (PCV). A PCV close to 100% indicates that individual variables explain most of the observed between-group variation. Lower PCV values suggest that the intersectional groupings explain outcomes beyond what individual variables capture alone.

## 3. Results

### 3.1. Sample Statistics

The study sample (Table 1) included 1154 women veterans with HIV, categorized across 17 strata. Most were aged 45–64 years, the majority were non-Hispanic Black, and 17% were classified as unhoused. Only 3 strata had fewer than 10 observations (none less than 5), indicating adequate sample sizes for most groups.

### 3.2. MAIHDA Models

For receipt of care, younger age, non-Hispanic White race, and being unhoused were associated with lower odds relative to the reference groups. For retention in care, younger age and non-Hispanic White race showed similar patterns of lower odds. For viral suppression, only younger age was significantly associated with lower odds. No significant differences were observed by Hispanic ethnicity or among older women. Results of the adjusted MAIHDA models can be found in Table 2.

Across all outcomes, VPCs in the null models were low, 2.4% for receipt of care, 3.0% for retention in care, and 1.8% for viral suppression. This suggests that most outcome variance occurred at the individual-level rather than between strata. In fully adjusted models, VPCs were reduced to zero, indicating that the model explains the observed variance in our dataset. PCV was 100% across all outcomes, suggesting that the fixed-effect covariates explained the modest between-stratum variance observed in the null models.

Across the 17 strata, predicted probabilities varied. For receipt of care, the highest predicted percentages clustered among housed, midlife or older, Hispanic (any race) and non-Hispanic Black women (ranging from 76% to 80%). The lowest estimates were observed among younger, unhoused, non-Hispanic White women (44–55%).

**Figure 1:**
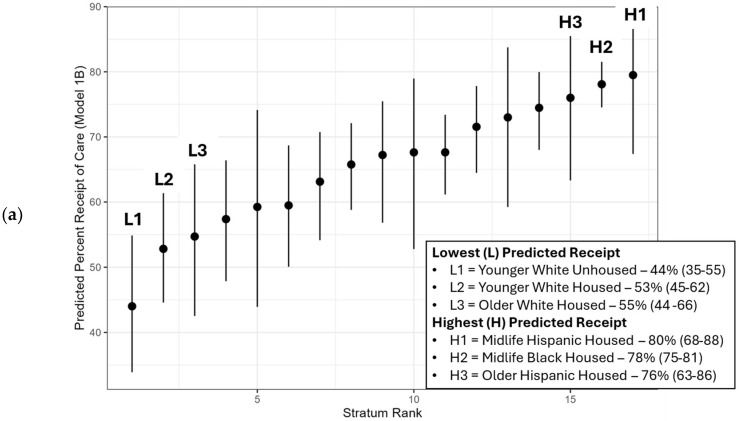

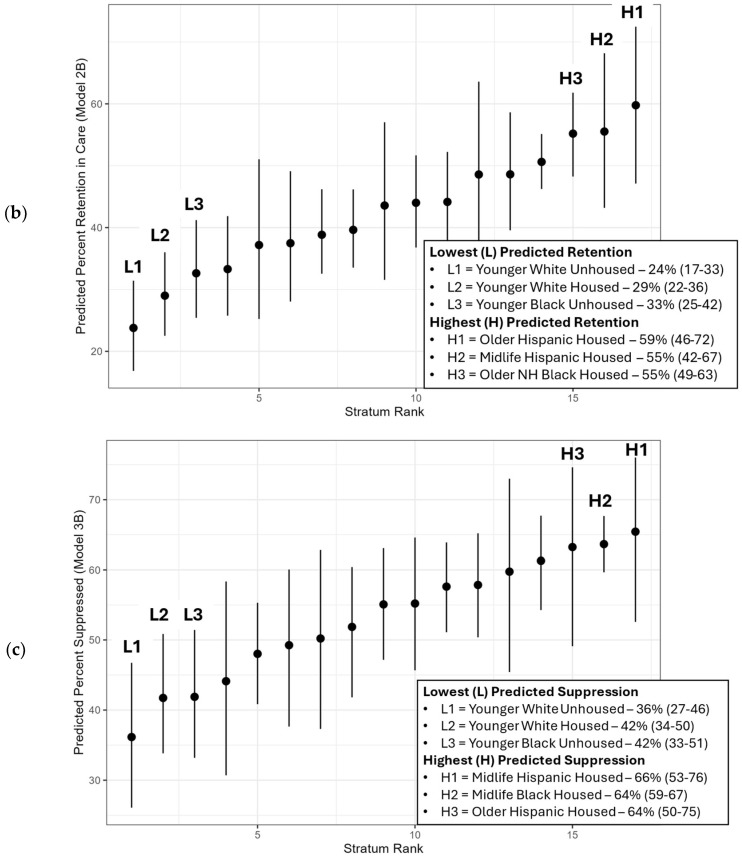
Predicted Percent Outcomes by Stratum Rank. Plots show predicted percent outcomes with 95% confidence intervals across 17 ranked strata, from mixed-effects logistic regression models adjusting for age, race/ethnicity, and housing status. Stratum rank is ordered from lowest to highest predicted outcome. Younger = <45 years; midlife = 45–64 years; older = 65+ years. (**a**) Receipt of Care; (**b**) Retention in Care; (**c**) Viral Suppression.

Retention in care followed a similar pattern, with the highest performing strata consisting of housed, midlife and older Hispanic and non-Hispanic Black women (55–59%). The lowest retention probabilities were observed among younger non-Hispanic White and Black women, regardless of housing status (24–33%). For viral suppression, the highest strata again included older and midlife, housed, Hispanic and non-Hispanic Black women (64–66%). In contrast, the lowest suppression probabilities were observed among younger, unhoused, non-Hispanic White and Black women (36–42%). Figure 1 summarizes the patterns of highest- and lowest-ranking strata by predicted outcome, with 95% CIs.

## 4. Discussion

To our knowledge, this is the first application of the MAIHDA framework to examine HIV care outcomes among women veterans in the VHA. Our results reveal additive patterns at the intersection of age, race/ethnicity, and housing status. In this study, observed patterns were generally consistent with broad HIV care trends documented in both civilian and veteran populations [4,29]. Prior research has shown that younger, unhoused individuals face greater challenges with retention in care and viral suppression, reflecting structural and life-stage barriers to sustained engagement. Our findings extend this literature by demonstrating how these patterns persist within an understudied, national cohort of women veterans. Also, by examining how these social positions operate jointly within a large integrated healthcare system.

Homelessness among women veterans is often linked to distinct gendered pathways, including military sexual trauma (referring to sexual assault or harassment experienced during military service), intimate partner violence, and post service reintegration challenges. This highlights the importance of tailored, trauma-informed approaches [30,31]. This may be particularly relevant for younger, unhoused women veterans in our sample who consistently had the lowest predicted probabilities across all outcomes.

Notably, our study found that Black and Hispanic women veterans demonstrated higher predicted retention and suppression than civilian trends suggest [2]. This finding contradicts patterns observed in the general population, where White women are more likely to achieve optimal HIV care outcomes than Black and Hispanic women. One possible explanation is that VHA system-level supports, such as integrated disease management protocols, telehealth and connected care services, and VHA-supported travel and housing assistance, may buffer disparities commonly observed outside the VHA [32]. For example, in conjunction with housing placement support, the VHA delivers care through coordinated wraparound services that integrate primary care, mental health treatment, case management and social support.

Although individual-level interventions may address observed disparities, the social positions examined in this study likely reflect broader structural barriers that cannot be resolved without system-level solutions. These include limited clinic hours that conflict with work schedules or caretaking responsibilities and challenges navigating health care while unhoused. Importantly, small VPC values do not imply poor model fit; rather, they indicate that most variation exists at the individual level, common in health services research where unmeasured factors (e.g., patient trust, stigma, care preferences) influence care experiences and sustained engagement. This underscores how traditional fixed-effects models capture independent associations but do not evaluate whether combinations of social positions compound risk. MAIHDA remains useful for explicitly testing whether intersectional strata help explain disparities in care outcomes. Ultimately, the absence of residual between-stratum variance in adjusted models does not diminish the value of an intersectional framework. It also does not rule out other structural factors (e.g., supportive social networks, health literacy, housing policies) that may contribute to the observed outcomes in our study [28,33].

### 4.1. Strengths and Limitations

A key strength of this study is its ability to highlight how multiple social positions operate simultaneously. Traditional fixed-effects models or single-variable approaches risk overlooking important intersectional patterns. In contrast, MAIHDA captures additive and multiplicative relationships without the instability that is common when including multiple interaction terms [21].

Despite these strengths, this study is not without limitations. Although MAIHDA is designed to handle small strata, including those with fewer than 10 individuals, estimates from very small groups (strata for Hispanic women, any race) should be interpreted cautiously due to potential instability and limited generalizability [27,28]. In this study, only 3 of the 17 strata had fewer than 10 individuals, suggesting that most stratum-level estimates were sufficiently stable.

Given the observational retrospective cohort study design, causality cannot be inferred. Although the study used temporally ordered data, the standard MAIHDA approach is cross-sectional in structure. Additionally, this study and method do not model cumulative or time-dependent exposures such as time on ART [27,34]. As a result, we could not account for the length of time on ARTs.

Housing status in our study is an administrative proxy and was identified using clinic stop codes tied to VHA homeless services. Some individuals coded as “unhoused” may already be transitioning into housing, while others experiencing housing instability but not seen in these clinics may have been missed, leading to possible misclassification or underreporting. Because our data does not distinguish between types of homelessness (e.g., sheltered, unsheltered, transitional housing), we were unable to analyze differences among these subgroups.

Race and ethnicity are intended to be patient self-report. This may also be captured by clinicians and staff and may not fully represent a veteran’s self-identification of their race or ethnicity. To maintain statistical stability in our models, we excluded smaller racial groups. Women veterans with HIV in VHA care who are Asian, American Indian/Alaskan Native, and Native Hawaiian/Other Pacific Islander collectively represented less than 3% of our sample. We chose not to collapse these groups into an “other” category as doing so would obscure meaningful differences in lived experiences and undermine the intersectional utility of MAIHDA as a framework. Finally, the social positions included in this study do not capture the full range of identity characteristics that may influence HIV care outcomes. Therefore, important aspects of intersectional identity characteristics (e.g., sexuality, marital status, rurality, etc.) may be overlooked.

### 4.2. Implications and Future Directions

These findings have important implications for both research and practice. First, they provide evidence that younger and unhoused women consistently showed lower predicted probability of care engagement and viral suppression, highlighting the need for interventions that address individual-level barriers rather than uniform-group-based solutions. Second, they reinforce the value of using stratified metrics within the VHA to identify and address group-specific barriers to care. However, because the PCV was 100%, the intersectional strata did not explain outcome differences beyond the included individual-level characteristics. Third, they demonstrate the feasibility and utility of applying MAIHDA in VHA research.

Future research should expand on these findings by comparing outcomes across VHA-only, VHA-paid community care, and civilian systems to understand how varying system-level infrastructure shape engagement and outcomes. Strength-based mixed-methods studies integrating qualitative interviews could reveal why some groups achieve better outcomes and how their care preferences may differ from lower-performing groups. For example, younger women veterans may benefit from flexible care delivery models that better accommodate their needs. Accommodations may include evening and weekend clinic hours, automated reminders, and other social supports such as childcare reimbursement or on-site childcare. These patient-centered adaptations may be especially important for improving engagement and respecting individual needs and preferences.

Future work could also examine how community-level supports and social cohesion are associated with HIV care outcomes. For example collective social networks and informal support systems may vary across groups and influence engagement beyond mere availability of services, individual resources, or health literacy [35,36]. Additionally, expanding intersectional models to include mental health, rurality, and care contexts (VHA and non-VHA care) and applying emerging approaches such as longitudinal or cross-classified MAIHDA models would capture the complexity of HIV care inequities better.

## 5. Conclusions

In conclusion, this study provides new insights into disparities in HIV care engagement among women veterans, identifying individual-level differences and variation in predicted outcomes at the intersection of age, race/ethnicity, and housing status. By applying multilevel modeling, we offer a nuanced understanding of where care gaps remain and which groups may benefit from focused interventions. Although individual characteristics explain most variation, these findings reinforce the need for tailored, patient-centered strategies that move beyond one-size-fits-all care. Continued use of intersectional analytic approaches can inform VHA and other health systems in designing more equitable care models that better serve populations facing compounding structural barriers to care. Even within an integrated system designed to reduce disparities, age and housing status remain strong predictors of suboptimal outcomes. This suggests that universal access alone may not be sufficient to achieve equitable HIV care outcomes among women veterans.

## Figures and Tables

**Table 1:** Sample Characteristics (N = 1154).

Variable	Category	n	%
Age Group (years)	Younger: <45	233	20%
	Midlife: 45–64	696	60%
	Older: 65+	225	19%
Race/Ethnicity	Non-Hispanic Black	821	71%
	Non-Hispanic White	271	23%
	Hispanic (any race)	62	5%
Housing Status	Housed	954	83%
	Unhoused	200	17%

**Table 2:** Parameter Estimates for Fully Adjusted Logistic MAIHDA Models of Receipt, Retention, and Viral Suppression Among Women Veterans in VHA care (N = 1154).

	Receipt of Care OR (95% CI)	Retention in Care OR (95% CI)	Viral Suppression OR (95% CI)
Fixed Effects: Regression Coefficients			
Intercept	**3.59** **(2.92–4.40)**	**1.03** **(0.86–1.22)**	**1.76** **(1.47–2.10)**
Age <45	**0.53** **(0.39–0.74)**	**0.62** **(0.45–0.84)**	**0.53** **(0.39–0.71)**
Age 65+	0.82 (0.58–1.15)	1.20 (0.88–1.62)	0.90 (0.66–1.22)
Hispanic (any race)	1.08 (0.60–1.94)	1.21 (0.71–2.05)	1.09 (0.64–1.87)
Non-Hispanic White	**0.58** **(0.44–0.78)**	**0.64** **(0.48–0.85)**	0.77 (0.59–1.02)
Unhoused	**0.70** **(0.50–0.97)**	0.77 (0.56–1.05)	0.78 (0.58–1.07)
Random Effects: Variances			
Stratum-level	0	0	0
Summary Statistics			
VPC	0%	0%	0%
PCV	100%	100%	100%

**Footnotes:** OR = Odds Ratio; CI = Confidence Interval; VPC = Variance Partition Coefficient; PCV = Proportional Change in Variance. Reference categories: Age group = 45–64; Race/Ethnicity = Non-Hispanic Black; Housing status = Housed. VPC calculated as: τ_00_/(τ_00_ + π^2^/3), where τ_00_ is the random intercept variance and π^2^/3 ≈ 3.29 for logistic models. Bolded estimates indicate statistical significance at *p* < 0.05.

## Data Availability

The data used in this study are not publicly available due to VHA privacy and confidentiality requirements. Deidentified data may be made available upon request with permission from the VHA.

## Abbreviations

The following abbreviations are used in this manuscript:

ART	Antiretroviral Therapy
FY22	Federal Fiscal Year 2022 (October 2021–September 2022)
FY23	Federal Fiscal Year 2023 (October 2022–September 2023)
HIV	Human Immunodeficiency Virus
ICD-10	International Classification of Diseases, Tenth Revision
MAIHDA	Multilevel Analysis of Individual Heterogeneity and Discriminatory Accuracy
PCV	Proportional Change in Variance
PrEP	Pre-Exposure Prophylaxis
VA	Veterans Affairs
VHA	Veterans Health Administration
VPC	Variance Partition Coefficient
